# Autologous cell-coated particles for the treatment of segmental bone defects—a new cell therapy approach

**DOI:** 10.1186/s13018-019-1219-5

**Published:** 2019-07-01

**Authors:** Dror Ben-David, Bettina Fishman, Guy Rubin, Atara Novak, Ilana Laevsky, Avinoam Kadouri, Yasmin Nishri Katz, Ora Burger, Asaph Zaretsky, Noam Bor, Ephraim Tzur, Shai Meretzki, Nimrod Rozen

**Affiliations:** 1 Bonus BioGroup Ltd, Matam Advanced Technology Park, 31905 Haifa, Israel; 2 Faculty of Medicine, Technion-Israel Institute of Technology, Haifa, Israel; 3 Department of Orthopedics, Emek Medical Center, Afula, Israel; 4 Department of Oral and Maxillofacial Surgery, Assaf Harofeh Medical Center, Zerifin, Israel

**Keywords:** Bone, Segmental bone defect, Stem cell

## Abstract

**Background:**

Adipose tissue-derived mesenchymal stem cells (AT-MSCs) are one of the most potent adult stem cells, capable of differentiating into bone, cartilage, adipose, muscle, and others. An innovative autologous AT-MSC-derived cell-based product (BonoFill-II) for bone tissue regeneration was developed to be suited as a bone graft for segmental bone defects.

**Methods:**

BonoFill-II was transplanted into 8 sheep with 3.2-cm full cortex segmental defect formed in the tibia. Bone regeneration was followed by X-ray radiographs for 12 weeks. At experiment termination, the healed tibia bones were analyzed by computed tomography, histology, and mechanical tests.

**Results:**

Our results indicate that one dose of BonoFill-II injectable formula led to an extensive bone growth within the transplantation site and to a complete closure of the critical gap in the sheep’s tibia in a relatively short time (8–12 weeks), with no inflammation and no other signs of graft rejection. This new and innovative product opens new prospects for the treatment of long bone defects.

**Conclusions:**

Injection of BonoFill-II (an innovative autologous cell therapy product for bone tissue regeneration) into a critical size segmental defect model (3.2 cm), generated in the sheep tibia, achieved full bridging of the gap in an extremely short period (8–12 weeks).

## Introduction

Bone defects due to trauma, infections, and skeletal diseases represent a major challenge to clinicians, from which, tibial fractures are the most common of all long bone fractures [1]. In the majority of simple fractures, the body’s self-healing mechanism is able to repair trauma-caused defects through a very well-studied healing cascade. The bone healing process is margined by a combination of osteoconduction—a material acting as a scaffold for the new bone to grow into—and cellular activity performed by different cell types including early mesenchymal cells and osteoprogenitors allowing osteoinduction—a combination of signals and cells promoting the newly generated bone. However, there are medical conditions where this process is severely impaired and clinical intervention is critically required. Aging, trauma, tumor resection, developmental deformities, and infection can lead to significant bone loss and large defects with poor intrinsic healing potential [2].

The gold standard solution is the use of autologous bone graft [3–5]. These grafts do not cause immunoreaction and act as live growing bone with all the osteoconductive and osteoinductive properties needed for healing. The autograft is typically obtained from either the anterior or posterior iliac crest, or when available, from the locally harvested bone. However, practically, this clinical practice is limited due to severe complications, such as donor site morbidity, limited mobility, and bone quantity and quality restrictions [5, 6]. For this reason, bone graft substitutes have been developed to enhance or replace the conventional autograft. The use of allograft or xenografts prevents these problems, but possesses an inferior bone quality, as well as risks of infections and immune response [5–8].

Mesenchymal stem cells (MSCs), capable of differentiation into bone, cartilage, adipose, muscle, tendon, ligament, and marrow stroma, have been found in a variety of tissues, including bone marrow, adipose, cord blood, placenta, and others [9]. Because of their relatively simple isolation techniques and their extensive differentiation potential and immune-modulation properties, MSCs derived from different tissues were introduced into the clinic for bone regeneration applications [10–12]. Mesenchymal-originating cells are responsible for creating and maintaining the skeletal architecture; these cells produce extracellular matrix proteins and are the regulators of matrix mineralization during bone formation and remodeling. Bone formation is characterized by a sequence of events starting with the commitment of MSCs to osteoprogenitor cells and their differentiation into pre-osteoblasts and then into mature osteoblasts capable of synthesis of the new bone matrix [12].

In vitro and in vivo studies have shown that adipose tissue-derived MSCs (AT-MSCs) are potent adult mesenchymal stem cells. Adipose tissue is relatively enriched with MSCs as compared to bone marrow tissue [13]. AT-MSCs normally reside in the stromal vascular fraction of the adipose tissue [14], making adipose tissue a promising cell source for tissue engineering products. Subcutaneous adipose depots are abundant and easily accessible in large quantities with a minimally invasive procedure (liposuction aspiration). Liposuction surgery is a well-tolerated and safe procedure yielding large quantities of aspirate. It is less expensive and less invasive in comparison with bone marrow aspiration for the purpose of stem cell isolation. AT-MSCs can be easily isolated by tissue digestion, followed by the outgrowth of the plastic adherent fraction from the primary isolated cell mixture [13, 15]. It has been shown that AT-MSCs-derived osteoprogenitors in vivo performance depends greatly on their ability to proliferate and differentiate on a 3D scaffold which serves as a carrier for the transplanted cells [16].

BonoFill-II, a bone grafting technology recently developed in our facility, introduces a novel cell-based autologous product for clinical use in the field of bone reconstruction. The developed therapy is based on cell culture derived from adipose tissue which grows on mineralized particles in a 3D bioreactor. During the cultivation period, which takes up to 20 days in total, the cells undergo mild osteogenic induction to become lineage committed. The cell-coated mineral particles are suspended in a semi-solid milieu to enable injectability of the product into well-defined bone defects. The autologous nature and osteogenic state of the cells, combined with the fact that the cells grow on separate micronic mineral particles, enables optimal conditions for the bone graft to be integrated and immunologically accepted upon transplantation. Besides their intended use in curing critical-size bone defects, these bone grafts may be employed to treat various disorders, including fractures, osseous defects from trauma, infection, tumors, and plastic and facial surgery reconstructions.

The human tibia is the most frequently broken long bone [1], often with significant bone loss. Those tibial defects can occur as a result of large tumor removal, trauma, or blast injuries [17]. Critical-sized bone defects (CSBD) are defined as “the smallest osseous defect in a particular bone and species of animal that will not heal spontaneously during the lifetime of the animal.” Those large bone defects represent a significant orthopedic challenge and pose a major clinical and socioeconomic problem, as they have a negative impact on patients’ quality of life due to consecutive reoperations and prolonged hospitalizations [18].

The sheep critical gap model is the most commonly used method to evaluate bone growth since the ovine tibia closely resembles that of the human tibia in terms of size, shape, and physical properties and is commonly used when studying human orthopedic diseases [17–19].

In this study, we demonstrate the ability of this new cell-based product to induce bone regeneration in a 3.2-cm size gap, in the sheep tibia.

## Methods

### Isolation and expansion of AT-MSCs

BonoFill-II is composed of highly viable adherent AT-MSCs extracted from human subject’s adipose tissue, adhered to and grown on mineral particles following suspension in a semi-solid milieu for transplantation.

Under the required regulatory approvals, human adipose tissue was obtained from liposuction procedures under local anesthesia. The tissue was washed with sterile phosphate-buffered saline (PBS; Biological Industries, Israel) to remove debris and red blood cells, then digested using 0.075% collagenase type I (Serva, Germany) for 30 min at 37 °C. Collagenase inactivation was done using an equal volume of Xeno-free medium (NutriStem MSC XF, Biological Industries, Israel). The processed tissue was centrifuged for 10 min at 1200 rpm, and the cellular pellet was re-suspended in Xeno-free medium. Cells were seeded for passage 0 cultivation at 150,000–200,000 cells/cm^2^ density. Medium change occurred every 3–4 days. Splitting along passages 0 to 4 using recombinant trypsin EDTA (Biological Industries, Israel) was performed when the culture reached 70–95% confluence with a seeding density of 3,000 cells/cm^2^. At passages 2–4, 2D-cultured AT-MSCs were harvested using recombinant trypsin and seeded on mineral (hydroxyapatite-based) particles. The cells were grown for up to 8 days in a closed stirred-based bioreactor in which they are osteogenically induced using BMP2. At the end of cultivation, the cell-coated mineral scaffold particles are washed from all ancillary remnants followed by suspension in a semi-solid milieu in a mixing and delivery system syringe. Final cell concentration per gram scaffold was 11 × 10^6^ cells (average), resulting in 5.6 × 10^6^ cells per ml (average).

### Surgical procedures

All surgical procedures and animal handling were approved by the Institutional Animal Care Committee of The Technion.

### Critical-sized gap surgery

Twelve 1-year-old female sheep (~ 57 kg) were operated in this study: eight in the test group and four in the control group. The animals were allocated to test groups randomly. Sheep were anaesthetized by 0.1 mg/kg bw Xylzine and 10 mg/kg bw Ketamin iv; induction was achieved by 4–6 mg/kg bw Propofol iv. Maintenance was performed by 1.5–2.0% isoflurane intubation + positive pressure ventilation of 100% oxygen, 15 breaths/min.

Continuous infusion of Fentanyl (5–10 mg/kg bw) was used as analgesic. To prevent infection, 20 mg/kg bw iv Cephazoline was injected before surgery and SID Ceporex (15 mg/kg bw) was applied for 10 days post-operation or longer, as necessary.

**Fig. 1:**
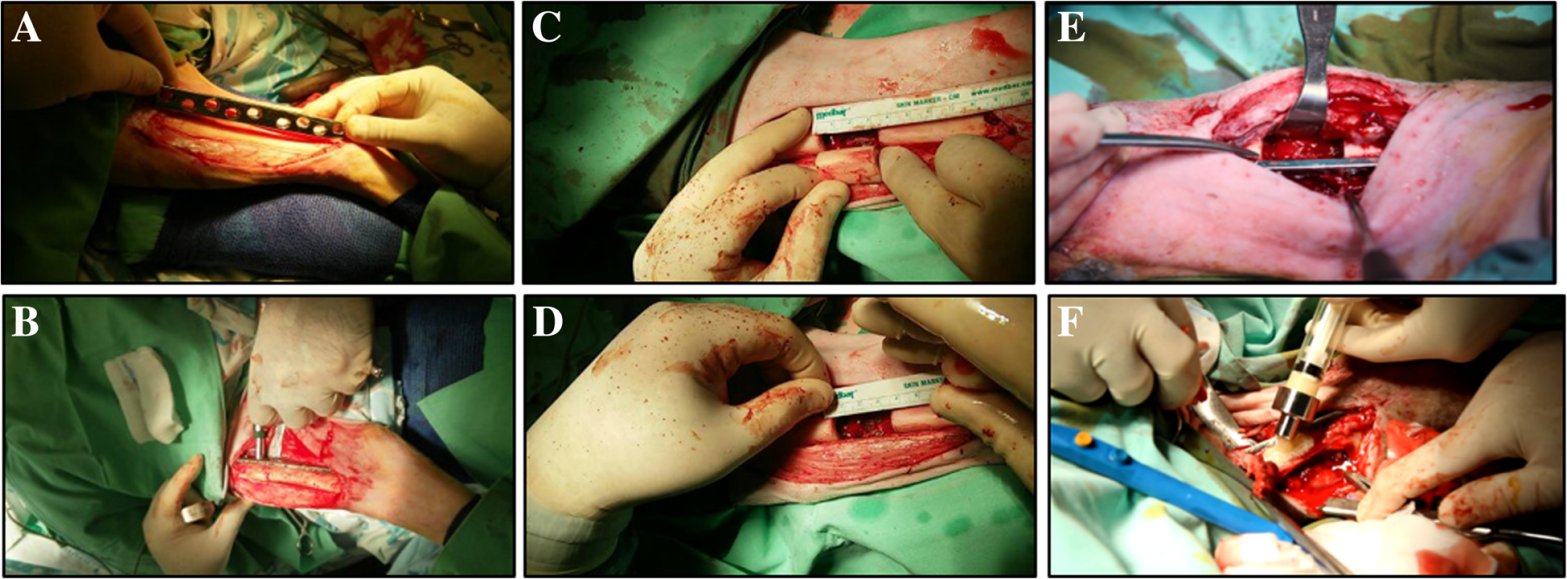
Surgical procedure. **a** To expose the bone, a longitudinal incision of 10–12 cm was made along the posterior–lateral aspect of the right tibia and a 4.5 stainless steel plate with 8–10 holes was adjusted to the morphology of the bone. **b** The plate was fixed to the posterior aspect of the tibia with 4.5 mm trans-cortical screws, 4 proximal screws, and 4 distal screws, leaving a central space of 3.5 cm. **c**, **d** A 3.2-cm cylinder was cut out of the tibia. **e** Two weeks following the first procedure, a limited incision just above the gap was performed. Then, a V wedge-shaped canal, about 5-mm deep and 3-mm wide, was cut across the regeneration scar tissue that filled the gap out all along the gap. **f** BonoFill-II injectable formulation (10 ml) was injected into the canal

The operation procedure was conducted as previously described by Rozen et al. [20] (Fig. 1). To expose the bone, a longitudinal incision of 10–12 cm was made along the posterior–lateral aspect of the right tibia, a few centimeters above the ankle joint and a few centimeters below the knee joint. A 4.5 stainless steel plate with 8–10 holes was then adjusted to the morphology of the bone by bending. The plate was fixed to the posterior aspect of the tibia with 4.5 mm trans-cortical screws, 4 proximal screws, and 4 distal screws, leaving a central space of 3.5 cm. To note, this method is commonly used in the treatment of human fractures, further confirming the size similarity between the ovine and human tibia [21]. After assuring the stability of the plate, a cylinder of 3.2 cm was cut out of the tibia, under continuous saline rinse. Afterwards, the wound was closed layer by layer. Post-surgery, the limb was fixed using a plaster cast for 2 weeks, excluding the ankle and knee joints for free movement. The sheep were examined on a daily basis for the first days after surgery, and food intake, behavior, and degree of lameness were closely observed.

Two weeks following the first procedure, sheep were anaesthetized as described above. A limited incision just above the gap was performed reaching the regeneration scar tissue that fills the gap.

A V wedge-shaped canal, about 5 mm deep and 3 mm wide, was cut out all along the gap. The piece removed was then trimmed-off its narrow edge leaving a topped-tailed tissue. Ten milliliters injectable formulation of BonoFill-II was injected into the canal. Alternatively, 10 ml of vehicle only (sham-transplanted control, containing a mixture of the mineral particles and the semi-solid milieu in ratio similar to BonoFill-II) was injected into the canal. Following injection, the canal was covered with the trimmed tissue and the wound was closed by layers as described above.

Upon termination of the experiment 12 weeks post-transplantation, the animals were sacrificed. The specimens were dissected and collected for general morphological evaluation, CT imaging, compression tests, and histological analysis.

### Evaluation of the healing process

The sheep’s health and well-being were monitored daily, and the transplantation site was analyzed once every 2 weeks using non-invasive X-ray imaging (CR+; GE Brivo 5555, lateral photo parameters MAS 2-1, KVP 75). Twelve weeks post-transplantation, upon sacrifice, the plates and screws were gently removed and tibia specimens approximately 6 cm long that included the healing gap and adjacent proximal and distal screw holes were excised and immersed in 10% natural buffered formalin for 7 days and then stored in 70% ethanol.

The specimens were subjected to morphological and mechanical evaluation, CT imaging, and histological analysis. The sham transplants and normal untreated bones served as controls.

### Micro-computed tomography

Whole specimens were scanned using a CT imaging system (Scannora 3DX, Soredex, Finland). Scanning was done using a 60-kVp energy setting and intensity of 10 mA, providing a resolution of 100 μm. The region of interest used for further analysis was between the last proximal and first distal screw holes.

The parameters analyzed included bone volume (BV/TV), bone surface (BS/TV), bone density, connectivity density, and polar moment of inertia (as a marker of the ability to resist torque strength).

### Histology

Central longitudinal midsagittal sections, 3-mm thick, were sawed out of transplanted sections. After decalcification (Rapid Decalcifier, Kaltek, Padova, Italy), the specimens were embedded in paraffin, and then subjected to histological processing. Six-micrometer-thick longitudinal and cross-section serial sections were stained with hematoxylin and eosin (H&E), for general histology evaluation of different areas in the gap. Masons trichrome staining was also performed to distinguish mature and immature bone tissue.

### Mechanical test

The treated bones were subjected to mechanical compression testing. Device used was the MTS - Model 312, load cell capacity 2.5 t (maximum load per sample was 25 kN).

In order to enable accurate replication of the experimental conditions in the numerical model, photographs and movies were taken before, during, and after the test (Allied Vision Technology 1.2-megapixel monochrome).

BonoFill-II-transplanted bone samples were compared to un-treated fixed bones. The samples were composed of 2-cm bone slices taken from the newly composed bone within the gap or from parallel segments in the control bones.

### Statistical analysis

Results are expressed as mean ± SEM. After testing for normality and equal variance, differences between vehicle and BonoFill-II-treated sheep were analyzed using *t* test (GraphPad Prism 5, USA). Differences were considered significant at *p* < 0.05.

## Results

BonoFill-II consists of multipotent, heterogeneous AT-MSCs. The cells are grown in high density on a 3D mineral scaffold in a bioreactor system that mimics their physiological microenvironment. The cells are seeded onto scaffold particles and undergo short osteogenic induction, before they are injected into the patient’s defected bone site, in order to induce optimal integration and commitment to the osteogenic lineage.

### Cell characterization

**Fig. 2:**
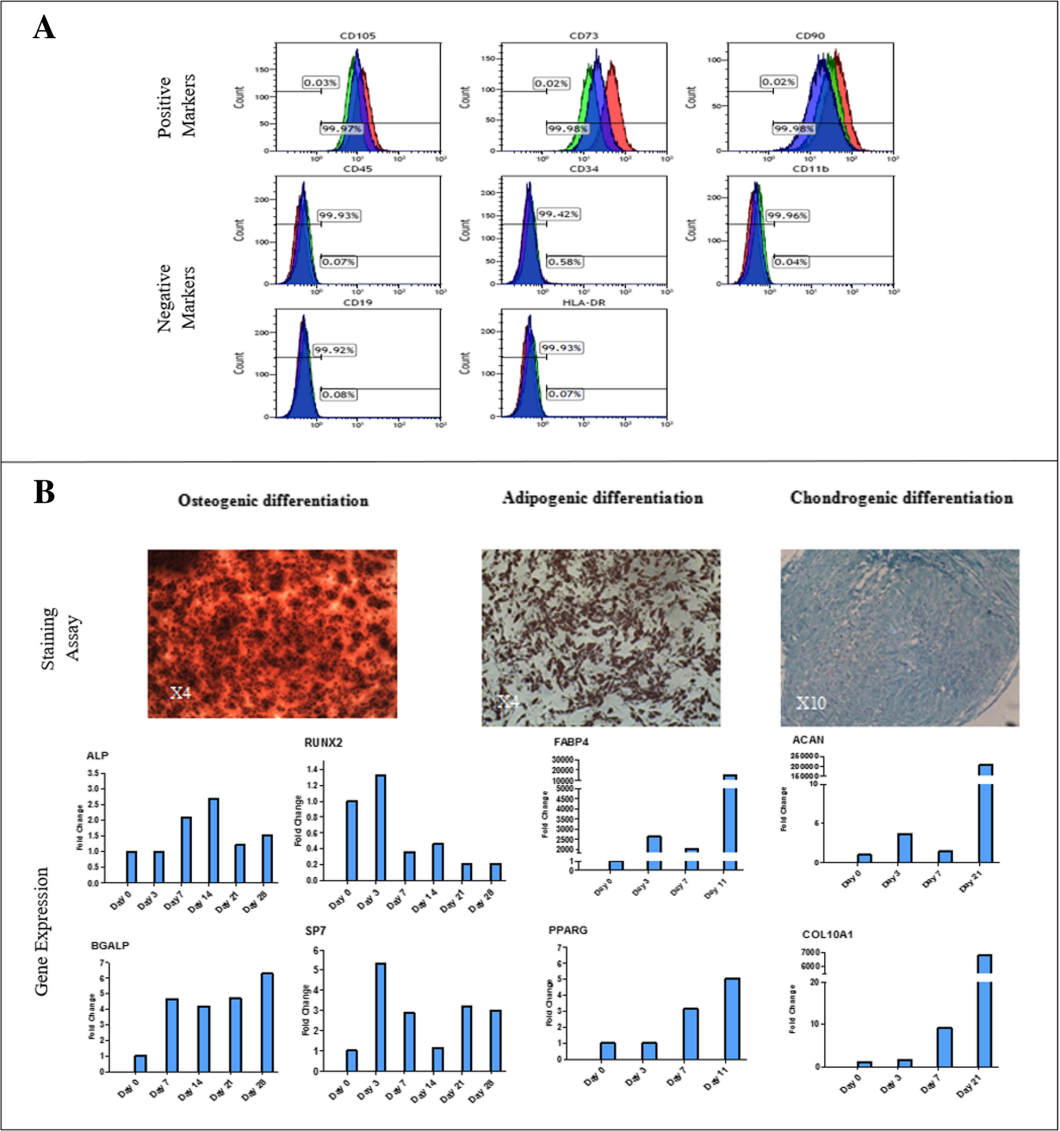
BonoFill-II characterization and osteogenic potential. **a** Expression profile of MSC surface markers of the isolated cells using FACS analysis (blue, red, and green graphs indicate different cell batches). **b** Multilineage differentiation potential of the isolated cells towards the osteogenic, adipogenic, and chondrogenic lineages as seen by specific gene expression and staining) (Alizarin Red S, Oil-red O, and Alcian Blue, respectively)

BonoFill-II culturing conditions include two stages. During the first growth stage, the AT-MSCs demonstrate classic MSC features, defined by common criteria, including (1) selective adherence ability and self-renewal; (2) positive staining for surface markers CD73, CD90, and CD105, and negative staining for surface markers CD11B, CD19, CD34, CD45, and HLA-DR (Fig. 2a); and (3) differentiation potential into the adipogenic, chondrogenic, and osteogenic pathways in vitro (Fig. 2b) [22].

Following the static growth period, the second growth phase is conducted in 3D stir tank bioreactor, which enhances osteogenic differentiation while supporting cell proliferation. This stage takes place in a closed system, in which the AT-MSCs are seeded on mineral particles and the culture incubation regime is changed to dynamic by a periodical stir of the system. Every 2–3 days, the conditioned growth medium is replaced with fresh medium. The shaker-based stirred tank bioreactor is a closed growth system, and all the growth medium replenishments are conducted using external accessories. Two days prior to the product assembly for transplantation, the culture medium is changed to osteogenic induction medium in order to start the osteogenic differentiation process. The cells’ osteogenic differentiation state before transplantation was evaluated by exploring the expression levels of osteogenic gene markers. Those markers include Osterix (SP7) and DLX5, which are key regulators of early osteogenic differentiation and are involved in the regulation of numerous osteoblasts-related genes, including osteocalcin, osteonectin, osteopontin, bone sialoprotein, and collagen type I [23], and ALP, a more advanced calcification activity marker [24] which is an important component in bone formation and is highly expressed in mineralized bone cells. Evaluation of advanced osteogenic differentiation capabilities of 3D-cultivated cells was done by exploring mineralization-related markers integrin-binding sialoprotein (IBSP) and osteopontin (SPP1).

**Fig. 3:**
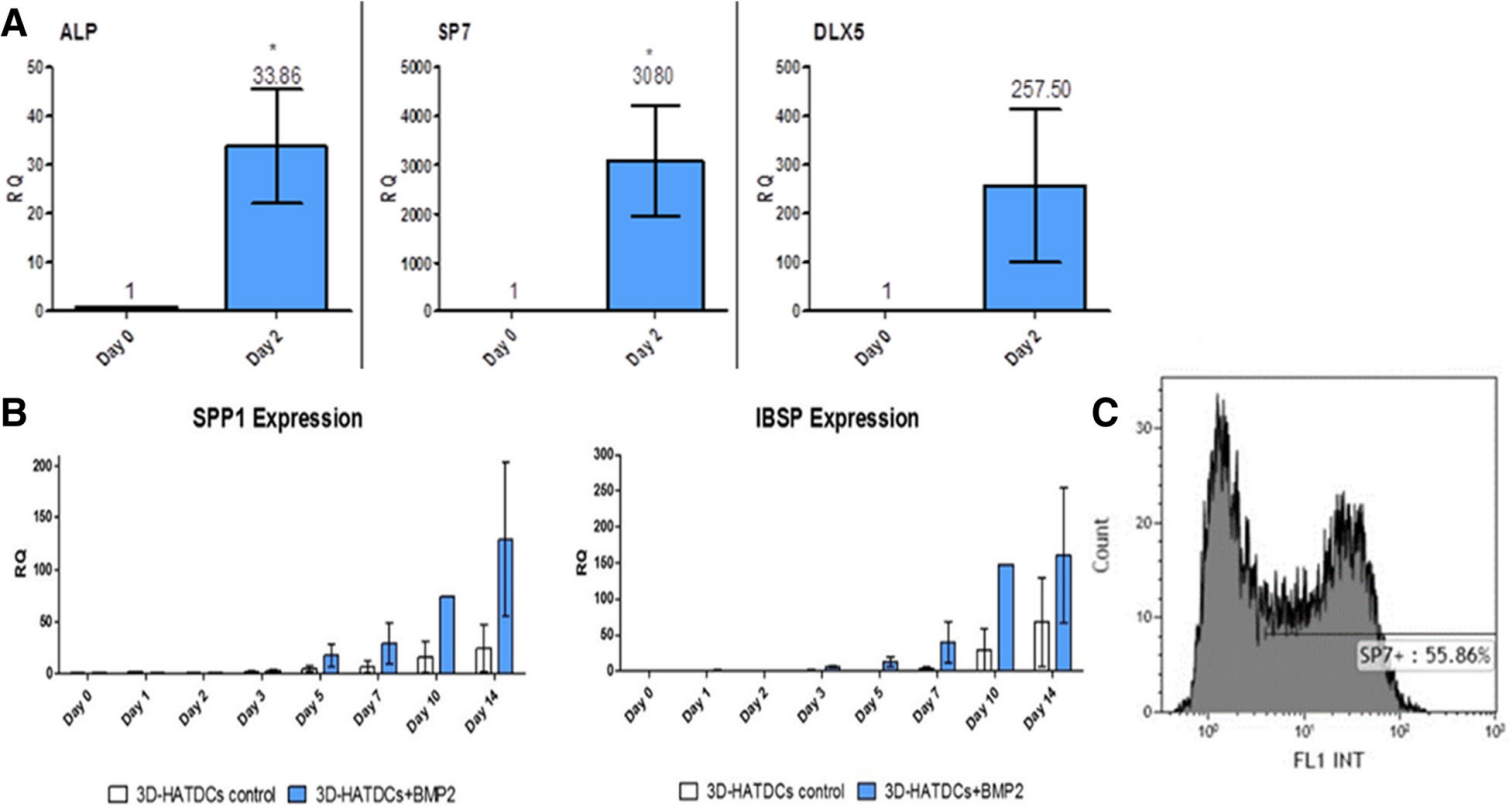
Osteogenic gene expression after osteogenic induction for 2 days. **a** qPCR analysis of early osteogenic markers ALP, SP7 (Osterix), and DLX5 gene expression at day 2 after osteogenic induction. **b** Time course of the late osteogenic markers SPP1 and IBSP. Values were normalized to day 0. Real-time analysis was done using ΔΔCt method and shown as fold change vs. control group and normalized to the RPLP0 housekeeping gene. Mean ± SEM (*n* = 6). **c** FACS analysis revealed that ~ 50% of the cells express SP7 protein after 2 days of osteogenic induction in the 3D system

In AT-MSCs treated with osteogenic induction medium, the expression of ALP, SP7, and DLX5 demonstrated upregulation on day 2 after induction (Fig. 3a). The late osteogenic markers *SPP1* and *IBSP* showed an increased expression starting on day 5 post-induction (Fig. 3b). Additionally, using FACS analysis, it was also found that ~ 50% of the cells express SP7 protein after 2 days of osteogenic induction in the 3D system (Fig. 3c).

### Critical-sized defect in the sheep tibia

Following the generation of a critical 3.2-cm gap in the sheep tibia, 8 sheep were transplanted with BonoFill-II (Fig. 1). Four sheep served as control and were transplanted with vehicle only. The sheep’s health and well-being were monitored daily, and the transplantation site was analyzed once every 2 weeks using non-invasive X-ray imaging. Substantial bone formation was observed in the gap area starting after 2 weeks post-surgery, leading to full bridging of the defect at experiment termination. This healing was observed in all of the experimental sheep transplanted with BonoFill-II. Two weeks post-transplantation and onward, the sheep grazed freely without limping and gradually gained 10–20% weight until the experiment termination. To be noted, the sheep’s original weight had no effect on the sheep’s behavior or on the end-point results.

### X-ray radiography follow-up

**Fig. 4:**
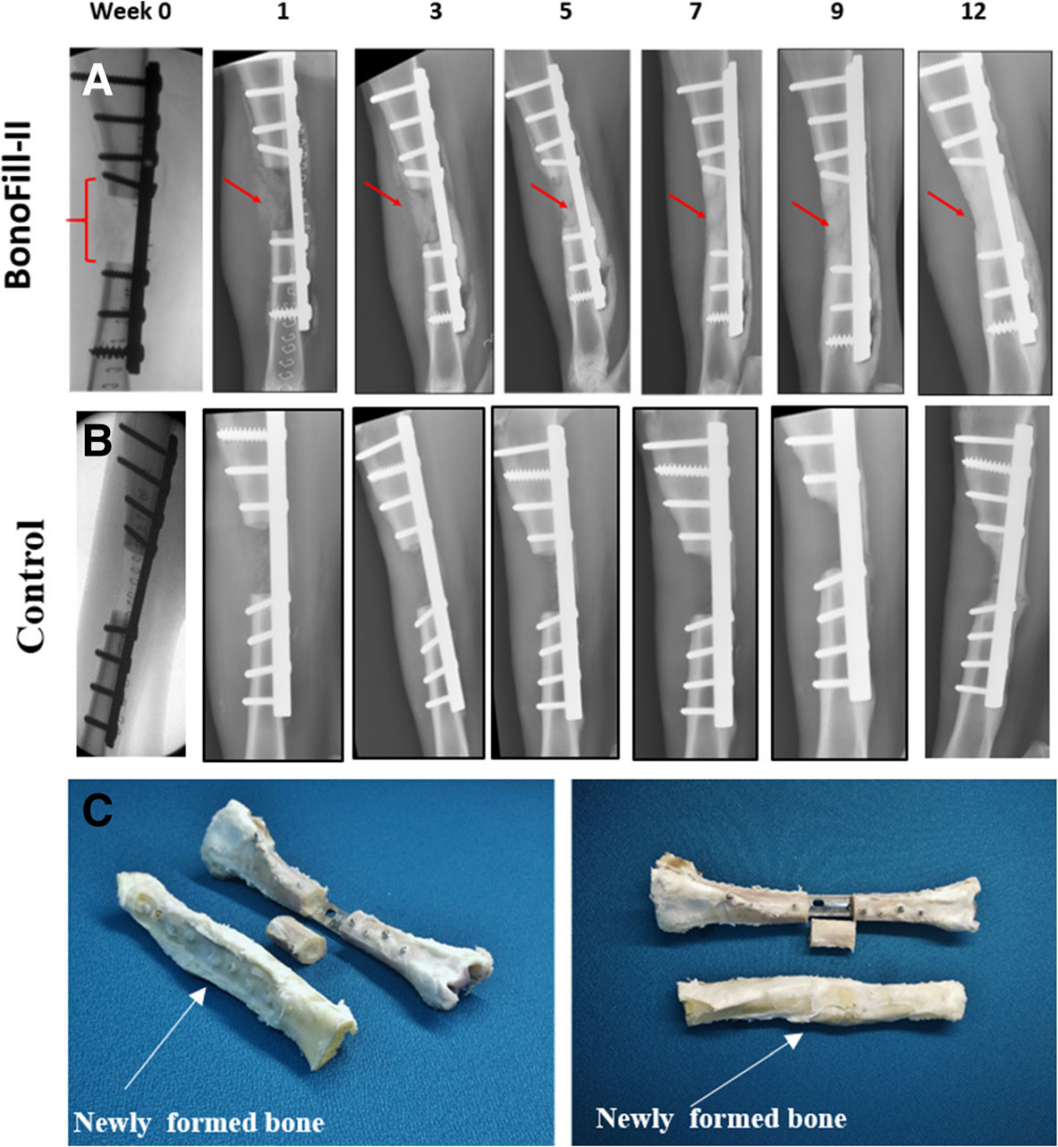
X-ray images and photographs of the treated tibia. **a** X-ray images of BonoFill-II-treated tibia. **b** Vehicle-treated tibia (control). Arrows indicate build-up of bone tissue in the gap. **c** Newly formed BonoFill-II-transplanted bones (front bones) after tibia extraction and models representing the 3.2-cm gap (rear bones)

X-ray follow-up showed that bone regeneration in the defects transplanted with BonoFill-II began 1–3 weeks following transplantation. The formation of the new bone increased significantly and continuously for up to week 12 post-transplantation, at which the gap was mostly filled and bone continuity was achieved. Representative X-ray follow-ups are demonstrated in Fig. 4.

### Micro-CT analysis

Micro-CT (μCT) analysis is presently implemented as the new gold standard method for quantifying bone microarchitecture [25]. This technique allows quantitative analysis of bone morphometric and density measurements of the bone formation within the defect.

**Fig. 5:**
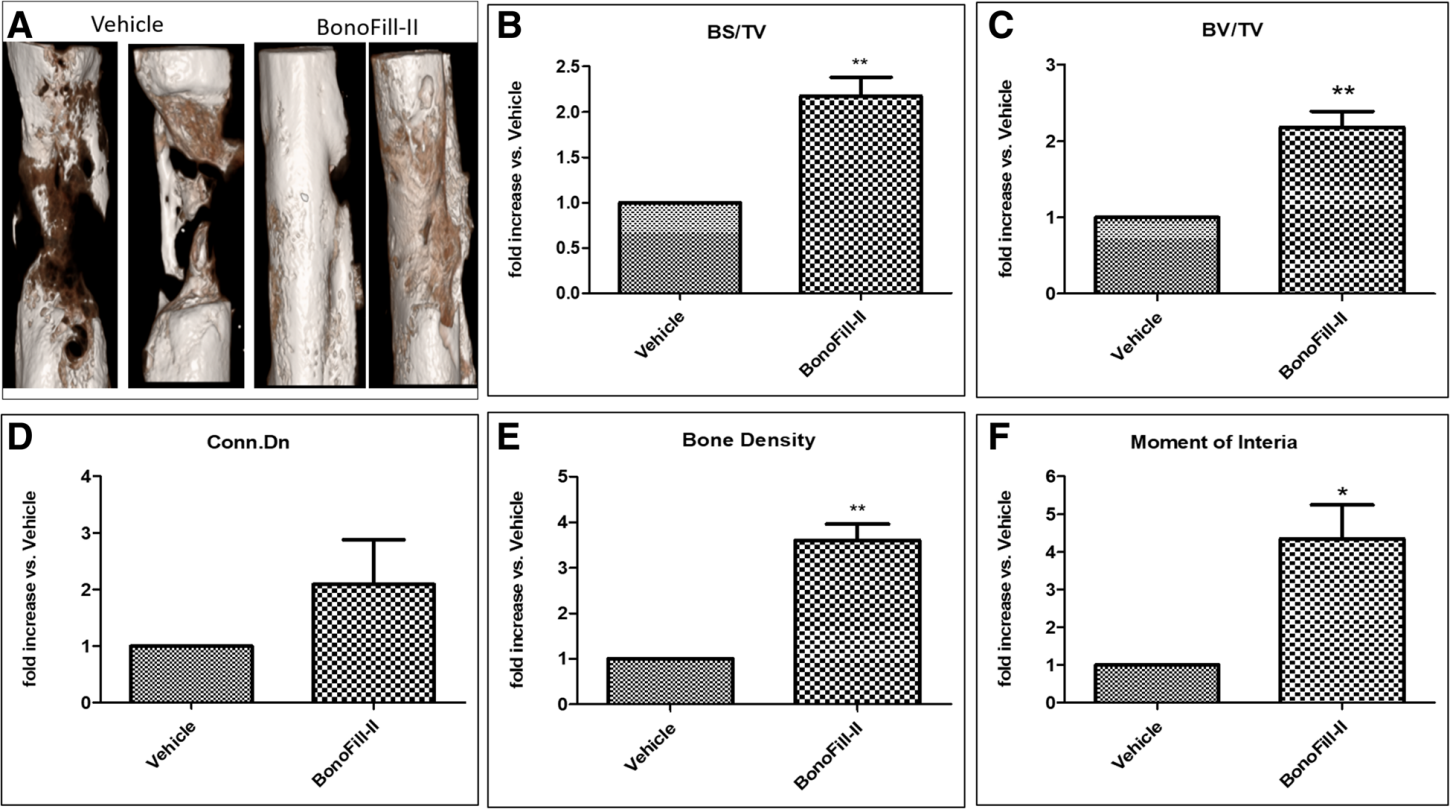
MicroCT analysis. **a** MicroCT scan at week 12 demonstrates full closure of the gap with a bone bridge in the BonoFill-II-treated group, compared to minimal bone growth in the vehicle group. **b**–**f** MicroCT analysis representing BonoFill-II-transplanted bones compared to vehicle-only-treated tibia. The parameters include BV/TV, BS/TV, bone density, and moment of inertia, respectively. All graphs represent fold of increase in the BonoFill-II-treated bones, compared to the vehicle-treated bones. *p < 0.05, **p < 0.01, ***p < 0.001

Micro-CT images, 12 weeks post-transplantation of treated tibia bones, showed substantially more bone formation and complete bridging inside the defect of the BonoFill-II-treated tibia, compared to the vehicle-transplanted group (Fig. 5a). Moreover, quantitative analysis of the micro-CT scans showed significantly superior results in the BonoFill-II-treated tibia compared to the vehicle-treated tibia. Those parameters included bone volume, bone surface within the tissue volume, bone density, connectivity density, and polar moment of inertia as a marker of the ability to resist torsion strength (Fig. 5b–f).

### Histological analysis

**Fig. 6:**
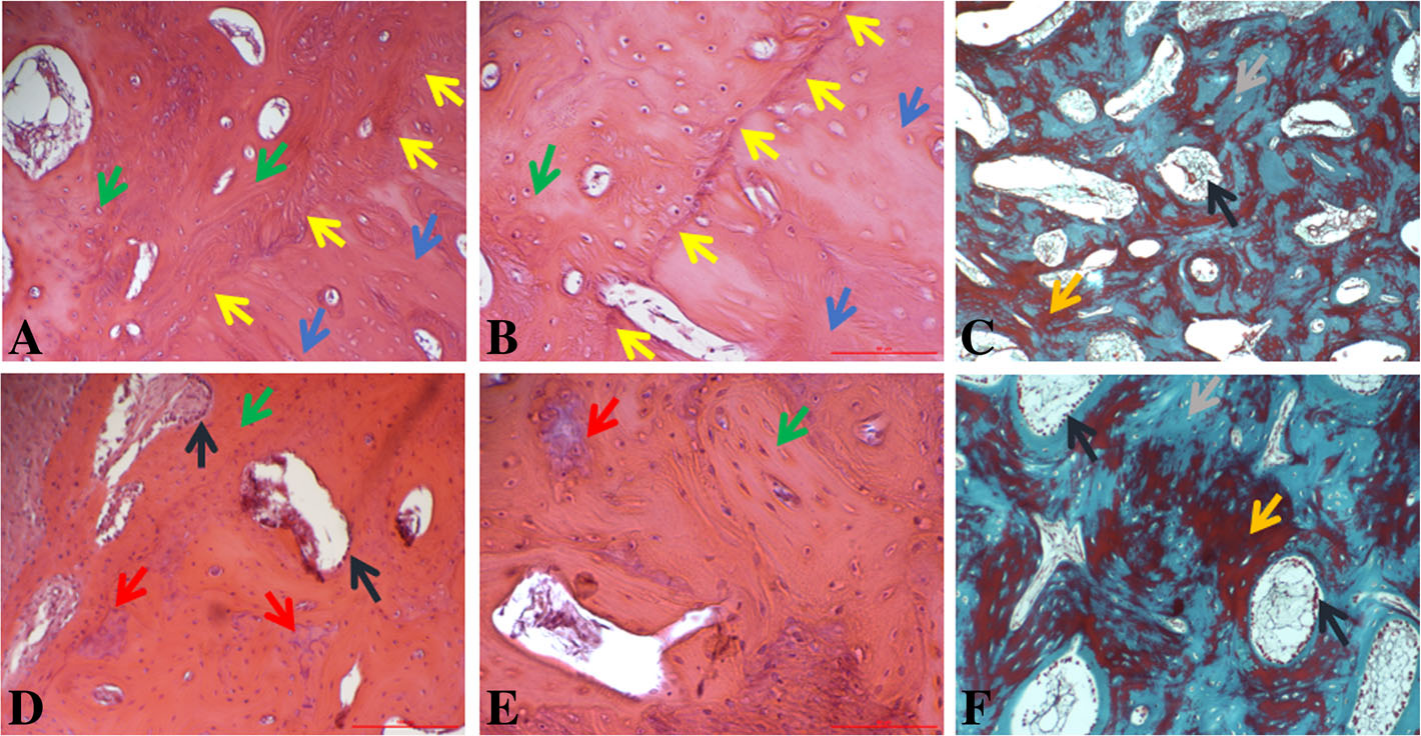
Histological analysis of areas within the bone gap. **a**, **b** These figures demonstrate the smooth integration of the new bone along the original bone (hematoxylin stain, × 10 and × 20, respectively). **d**, **e** Bone growth inside the gap area revealing compact bone arrangement of the newly formed bone and areas of maturating woven bone (hematoxylin stain, × 10 and × 20, respectively). **c**, **f** Masson’s trichrome stain demonstrating mineralized tissue with areas of premature bone- and marrow-like tissue (× 4 and × 10, respectively). Light blue arrows represent viable, original untreated bone; yellow arrows represent the transition between original and newly formed bone; green arrows represent newly formed compact bone; red arrows represent areas with chondrogenic activity indicating that endochondral ossification has not completed; and dark blue arrows mark areas with marrow-like areas with high osteoblastic activity

Histological analysis of the generated bone inside the gap 12 weeks post-treatment showed smooth integration of the new bone along the original bone and the formation of mostly compact bone within the gap with areas of woven bone (Fig. 6a, b). Masson’s trichrome stain shows that the generated bone tissue was extensively mineralized with some areas of immature tissue that will be mineralized due time (Fig. 6c, f). The newly formed bone was rich in chondrogenic and osteoblastic activity in areas resembling marrow tissue, indicating that endochondral ossification has not completed (Fig. 6d, e). Furthermore, many blood vessels were seen in the area of the new bone formation with some vessels crossing the original and new bone. Importantly, no evidence of immune reaction was seen.

### Mechanical tests

**Fig. 7:**
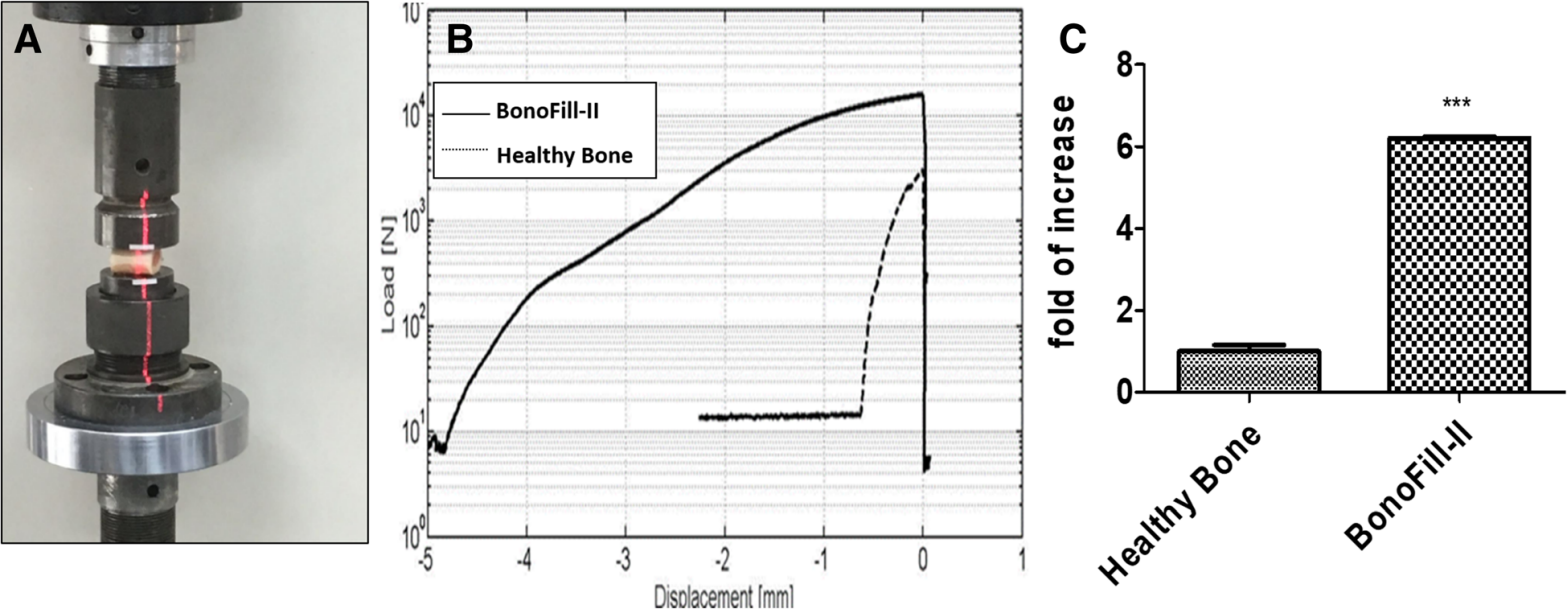
Compression tests performed on normal and BonoFill-II transplanted bones. a Compression test using the MTS loading machine - Model 312. **b** Representative BonoFill-II-transplanted bone sections and parallel normal bone; the graph indicates a load vs. displacement curve. **c** Load represented as fold of increase in BonoFill-II-treated bones compared to normal bones. ***p < 0.001

Compression of 2-cm sections of BonoFill-II-transplanted bones was compared to similar sections of normal bones using the MTS loading machine (Fig. 7a). Figure 7b displays a typical compression curve obtained using this experimental setup. At first, the force increases linearly followed by steep decrease of the curve due to material damage. At this point, cracks started to appear. Once the maximum load was reached, the specimen’s structure broke and the curve decreases. As seen in the Fig. 7b and c, about 7 times more load was needed in order to crack the BonoFill-II-transplanted bones, as compared to normal bones.

## Discussion

Bone reconstruction is usually performed by autologous bone grafting, which is considered the gold standard procedure, for sizeable bony injuries [2, 26, 27]. However, despite their prevalence, autologous bone grafting have major disadvantages, including operative and post-operative complications, morbidity at the donor site, scarcity of autologous bone, and poor bone quality [26]. Alternatively, various synthetic bone substitutes and biomaterials have been developed, but as for now, do not provide optimal bone regeneration efficiency [28].

BonoFill-II, an autologous bone graft, derived from the patient’s adipose tissue was developed in order to overcome the existing limitations of those current therapeutic approaches. BonoFill^TM^ consists of multipotent, heterogeneous AT-MSCs grown on mineral particles in a 3D bioreactor.

In this study, BonoFill-II was evaluated for its ability to induce bone regeneration and gap-bridging in a critical-sized bone defect of 3.2 cm in sheep tibia. Furthermore, the bone healing capabilities were compared to a vehicle-only control, which did not contain any cells.

The results of this study demonstrate that a single administration of BonoFill-II into the bone gap led to significant new bone formation within the defect area, which resulted in complete bone continuity within 12 weeks post-transplantation, as was corroborated by X-ray photography, CT scans, and histological analysis. Furthermore, there were no signs of post-operative inflammation or rejection in any of the animals. These findings correlate with previous studies demonstrating no evidence of inflammation or rejection after human MSC transplantation in mini pig or in sheep tibia models [26, 27], probably due to MSC low immunological activity [29, 30]. Notably, in the vehicle (containing no cells)-treated animals, the bone healing process failed to instigate and the gap remained mainly unchanged (Fig. 6).

The existence of a complete bone closure was further demonstrated using compression tests, showing a sturdy bone, stronger than the healthy control samples (the other healthy tibia of the sheep). Moreover, polar moment of inertia tests as shown in the CT analysis demonstrated that treated bones resist torque significantly more than control bones; this trend was also shown by Schoenau et al. [31]. Thus, the morphology of this newly formed bone corresponded to normal bone during fracture healing at the remodeling stage, which is the last stage of normal fracture healing and lasts months to years until the healing bone is restored to its original shape, structure, and mechanical strength, and is facilitated by mechanical stress placed on the bone. As the fracture site is exposed to an axial loading force, bone is generally laid down where it is needed and resorbed from where it is not needed. Adequate strength is typically achieved in 3 to 6 months [32].

Although the use of autologous MSCs transplants is being continuously studied in the field of bone tissue regeneration, the majority of studies did not use cell-seeded scaffold particles. The most frequently used techniques are mixture of cells and scaffold, cells injected onto the implant during the transplantation procedure [33, 34], and also cells injected weeks after the implantation of the scaffold [35]. Injecting only MSCs or bone-forming cells into the bone fracture gap was unsuccessful in inducing sufficient bone regeneration and consequently failed to close the larger segmental gap [36]. Although these methods show much potential and success in the treatment of modest segmental defects, large segmental defects require a more comprehensive approach. The abstention from using cell-seeded scaffolds is mainly attributed to the fact that cell growth within scaffold constructs requires complexed culturing techniques and entails many fallbacks as lack of homogenous growth and decreased viability in centric areas of large constructs [37, 38]. The novelty of our approach to overcome such limitations in large segmental defects is the usage of small mineralized carriers densely covered with cells which are injected into the defect site. The separate particles enable diffusion of sufficient nutrients during the initial period following transplantation and the fast penetration of blood vessels to facilitate viable and healthy bone growth. These mineralized particles serve as ossification seeds that connect together and lead to full segmental defect healing with healthy bone tissue, even up to 10 cm gaps (data not shown). Moreover, by injecting cell-coated particles, we ensure the remaining of the cells in the transplantation site and improve their survival rates.

## Conclusions

One dose of BonoFill-II injectable formula led to bone growth and to a complete closure of a critical gap in the sheep’s tibia in a relatively short time with no inflammation and no other signs of graft rejection.

This new and innovative treatment opens new prospects in the treatment of long bone defects. Based on this pre-clinical study, we believe that BonoFill-II can effectively regenerate bone tissue and bridge bone gaps in human subjects. For this purpose, suitable clinical studies should be designed and conducted.

## Data Availability

Data is available in Bonus BioGroup.

## Abbreviations

AT-MSCs: Adipose tissue-derived mesenchymal stem cells
CSBD: Critical-sized bone defects
H&E: Hematoxylin and eosin
MSCs: Mesenchymal stem cells
μCT: Micro-CT
